# Predictors of 15-year transitions across living and care settings in a population of Swedish older adults

**DOI:** 10.1093/ageing/afaf006

**Published:** 2025-01-26

**Authors:** Susanna Gentili, Amaia Calderón-Larrañaga, Debora Rizzuto, Adam Lee Gordon, Janne Agerholm, Carin Lennartsson, Åsa Hedberg Rundgren, Laura Fratiglioni, Davide Liborio Vetrano

**Affiliations:** Aging Research Center, Department Neurobiology, Care Sciences and Society, Karolinska Institutet and Stockholm University, Stockholm, Sweden; Aging Research Center, Department Neurobiology, Care Sciences and Society, Karolinska Institutet and Stockholm University, Stockholm, Sweden; Stockholm Gerontology Research Center, Stockholm, Sweden; Aging Research Center, Department Neurobiology, Care Sciences and Society, Karolinska Institutet and Stockholm University, Stockholm, Sweden; Stockholm Gerontology Research Center, Stockholm, Sweden; Academic Unit of Injury Recovery and Inflammation Sciences (IRIS), School of Medicine, University of Nottingham, Nottingham, and National Institute for Health and Care Research, London, UK; Aging Research Center, Department Neurobiology, Care Sciences and Society, Karolinska Institutet and Stockholm University, Stockholm, Sweden; Aging Research Center, Department Neurobiology, Care Sciences and Society, Karolinska Institutet and Stockholm University, Stockholm, Sweden; Swedish Institute for Social Research (SOFI), Stockholm University, Stockholm, Sweden; Stockholm Gerontology Research Center, Stockholm, Sweden; Aging Research Center, Department Neurobiology, Care Sciences and Society, Karolinska Institutet and Stockholm University, Stockholm, Sweden; Stockholm Gerontology Research Center, Stockholm, Sweden; Aging Research Center, Department Neurobiology, Care Sciences and Society, Karolinska Institutet and Stockholm University, Stockholm, Sweden; Stockholm Gerontology Research Center, Stockholm, Sweden

**Keywords:** care transitions, ageing, hospitalisation, nursing home placement, formal care, integrated care, personalised medicine, older people

## Abstract

**Objective:**

We aimed to investigate the association of sociodemographic, clinical and functional characteristics with the volume of transitions and specific trajectories across living and care settings.

**Methods:**

Using data from the Swedish National Study on Aging and Care in Kungsholmen study, we identified transitions across home (with or without social care), nursing homes, hospitals and postacute care facilities among 3021 adults aged 60+. Poisson and multistate models were used to investigate the association between sociodemographic, clinical and functional characteristics and both the overall volume and hazard ratios (HRs) of specific transitions.

**Results:**

Over 15 years, 720 (23.8%) participants experienced between 5 and 10 transitions, and 816 (26.7%) experienced >10 transitions across living and care settings. A higher number of transitions was observed in older participants with multimorbidity and slower walking speed. In contrast, cognitive impairment and disability were associated with a lower number of transitions. After hospital and postacute discharge, each additional year of age (HR range 1.06–1.08) and being a woman compared with being a man (HR range 1.35–4.38) increased the likelihood of discharge to home care. Multimorbidity (HR range 1.14–1.23) and slow gait speed (HR range 1.11–1.50) increased the risk of hospitalisation and home care after hospital discharge. Cognitive impairment raised the hazard of nursing home placement (HR range 1.99–2.15). Disability was associated with a higher hazard of nursing home placement after hospital discharge (HR range 2.57–3.07).

**Conclusions:**

Accounting for older adults’ whole journey across living and care settings, we identified transition-specific predictors and potential triggers that could be timely leveraged to better tailor care to older adults’ needs.

## Key Points

- Multimorbidity and slow gait speed increase the likelihood of hospital admission and home care.
- Cognitive impairment is associated with a higher likelihood of nursing home placement.
- Recognising the factors influencing specific and intense healthcare transition patterns might open to personalised prevention approaches.

## Background

‘Ageing in place’ means individuals should be facilitated to live in their homes and community safely, independently and comfortably, regardless of age, income or ability [1, 2]. However, delivering these objectives is challenging when working with older individuals with complex clinical, functional and psychosocial profiles who are exposed to frequent and recurrent transitions across different life and care settings [2, 3]. Fragmented care increases risk of hospitalisation, mortality and healthcare expenditure [4–8], poor treatment adherence and accelerated functional decline. Identifying older individuals at higher risk of frequent care transitions is therefore important [9–11].

Most previous studies have focused on specific care transitions. Several have investigated factors associated with frequent hospitalisation and rehospitalisation in older adults, with age, gender and disease burden proving significant [12, 13]. Risk factors for nursing home placement have also been investigated [14–17]. The complexity of older people’s journeys through the healthcare system, encompassing care from multiple providers, different healthcare settings and repeated transitions through the same setting (e.g. hospitals), has not been substantively studied [6, 7, 9–11].

Using a well-characterised cohort of Swedish older adults followed up to 15 years and employing Poisson and Markov models to account for the complexity of living and care setting transitions, we aimed to investigate the association of sociodemographic, clinical and functional characteristics with (i) the number of transitions across living and care settings (i.e. home, home care, nursing home and hospital) and (ii) specific trajectories across living and care settings, in Swedish older adults.

## Methods

### Study population

The Swedish National Study on Aging and Care in Kungsholmen (SNAC-K) provided the study data [18]. SNAC-K started in 2001, inviting 4590 older adults (60+) from 11 age cohorts; 3363 (73%) participated (Table S1 and Figure S1). Participants under 78 years of age were followed up every 6 years, while those over 78 years were followed up every 3 years (Figure S2). Additional details of SNAC-K can be found in Appendix 1. In our study, 342 individuals were excluded because of missing values across potential predictors (Table S2). The observation period began at baseline (2001–04) and was administratively censored in January 2017, except for participants who dropped out during follow-up, whose data were censored at the last follow-up date. Further details regarding sampling and design have been published previously [18].

### Sociodemographic characteristics

Sociodemographic data (age, gender, level of education and civil status) were fixed at baseline. Elementary school and high school, or university and above, were used to categorise the highest educational attainment. Participants’ civil status was dichotomised as partnered, meaning married or in a relationship, or unpartnered, meaning widowed, divorced or without a partner.

### Health and functional status assessment

Global cognitive performance was evaluated using the Swedish Mini-Mental State Examination (MMSE) and dementia diagnosis. MMSE scores range from 0 (severe dementia) to 30 (normal cognitive status) [19, 20]. According to O’Bryant *et al.*, a cutoff of 27 was used to detect cognitive impairment in highly educated older adults [21], with scores higher than 27 considered normal and ≤27 indicating cognitive impairment. Dementia was diagnosed through a three-step clinical procedure following Diagnostic and Statistical Manual of Mental Disorders (DSM-IV) criteria [22].

Diseases were ascertained based on a thorough assessment. Chronic diagnoses were classified into 60 categories using the International Classification of Diseases, Tenth Revision [23]. The details of these classifications have been reported previously [24]. Coexistence of three or more chronic diseases was defined as multimorbidity [24–26]. To minimise collinearity issues with the assessment of cognitive impairment, dementia diagnoses were not included in multimorbidity.

Gait speed (m/s) was measured by having participants walk 6 m at their chosen pace or 2.44 m if they reported slow walking (walking aids were allowed). Gait speeds of ≤0.8 and >0.8 m/s were classified as the presence or absence of walking impairment, respectively [27, 28].

Disability in Activities of Daily Living (ADL) was defined as at least one impairment in: bathing, dressing, toileting, transferring/mobility (ability to transition from a seated position to standing; as well as ability to get in and out of bed and walk) and eating [29].

Disability in Instrumental Activities of Daily Living (IADL) was defined as at least one impairment in: food shopping, money management, housework, using a phone or taking public transit [30].

### Outcomes: living and care setting

Home was defined as the usual living setting, either owned or rented. Information about living places was collected at baseline and follow-up visits.

Home care was defined as living at home and receiving formal care services from professionals. This included domestic duties or personal care provided by municipalities, which cover costs of formal care [31, 32]. In this study, we only considered the presence or absence of formal care at baseline and follow-up visits.

Information about nursing home residency was gathered using a self-report questionnaire at each study wave (baseline and follow-up) [32, 33].

A hospital or postacute/rehabilitation care episode was defined as an admission lasting at least one full night. Postacute/rehabilitation was operationalised using hospital codes to identify rehabilitation facilities. This information was retrieved from the National Patient Register between 2001 and January 2017 and was available for the entire study sample [34, 35].

The Swedish Cause of Death Registry (Dödsorsaksregistret) was used to determine the date of all-cause mortality during the study period (2001–17) [36, 37].

### Statistical analysis

Baseline characteristics were summarised using mean and standard deviation (SD), and counts and percentages, for continuous and categorical variables, respectively. All sociodemographic, clinical and functional characteristics were fixed at baseline. Stepwise Poisson regression models were used to determine the incidence rate ratio (IRR) of the total number of transitions associated with each predictor. The IRR represents the relative change in the incidence rate of the event for a single unit change in the baseline characteristic. A Markov multistate reversible model was used to assess the likelihood of transitioning across living and care settings based on baseline demographic, clinical and functional characteristics (Figure S3). This model accounts for reversibility (i.e. where transitions between different states can occur in both directions) and considers multiple outcomes simultaneously (i.e. hospitalisation, nursing home placement, home with and without use of formal care, and mortality). Appendix 2 provides additional details on the Markov multistate reversible model. The total number of transitions between living and care settings was visualised using Sankey plots. These illustrate the flow of participants across different transitions and don’t account for the time dimension. The mean length of stay and expected number of visits to each state were estimated. Hazard ratios (HRs) and 95% confidence intervals (CI) were calculated to assess the association between each predictor and transition pattern [38]. Transition types with fewer than 20 events (e.g. post-acute—nursing home) were excluded for more precise parameter estimation. All analyses were performed in R using glm2 for Poisson regression [39] and MSM for the Markov multistate model [38].

## Results

Baseline sociodemographic, clinical and functional characteristics of the 3021 study participants by living setting are reported in Table 1. At baseline, 2614 (86.5%) were living independently at home, 329 (10.8%) were living at home in receipt of home care services and 78 (2.7%) were living in nursing homes.

**Table 1:** Baseline sample characteristics according to the living setting.

	Living at home		Living in nursing home	Total
	Without formal care	With formal care		
	*n* (%)	*n* (%)	*n* (%)	*N* (%)
Sample	2614 (86.5)	329 (10.8)	78 (2.7)	3021 (100.0)
Age (years)				
Mean ± SD	71.8 ± 9.7	86.3 ± 7.9	89.6 ± 7.5	74.7 ± 11.2
60–69 years	1252 (47.9)	11 (3.3)	1 (1.4)	1264 (41.8)
70–79 years	822 (31.5)	53 (16.2)	6 (7.6)	881 (29.2)
80–89 years	413 (15.8)	114 (34.6)	16 (20.5)	543 (17.9)
≥90 years	127 (4.8)	151 (45.9)	55 (70.5)	333 (11.1)
Gender				
Men	1022 (39.1)	66 (20.1)	14 (17.9)	1102 (36.5)
Women	1592 (60.9)	263 (79.9)	64 (82.1)	1919 (63.5)
Civil status				
Partnered	1378 (52.7)	45 (13.7)	15 (19.2)	1438 (47.6)
Unpartnered	1236 (47.3)	284 (86.3)	63 (80.8)	1583 (52.4)
Education				
Elementary school	351 (13.4)	109 (33.1)	27 (34.6)	487 (16.1)
High school/University	2263 (86.6)	220 (66.9)	51 (65.4)	2534 (83.9)
Cognitive impairment^a^				
No	2438 (93.3)	182 (55.3)	11 (14.1)	2631 (87.1)
Yes	176 (6.7)	147 (44.7)	67 (85.9)	390 (12.9)
Multimorbidity (3+ diseases)^a^				
No	936 (35.8)	17 (5.2)	16 (20.5)	969 (32.1)
Yes	1678 (64.2)	312 (94.8)	62 (79.5)	2052 (67.9)
Slow gait speed (<0.8 m/s)				
No	2199 (84.1)	48 (14.6)	7 (9.0)	2254 (74.6)
Yes	415 (15.9)	281 (85.4)	71 (91.0)	767 (25.4)
≥1 Impaired ADL^a^				
No	2580 (98.7)	252 (76.6)	15 (19.2)	2847 (94.2)
Yes	34 (1.3)	77 (23.4)	63 (80.8)	174 (5.8)
≥1 Impaired IADL^a^				
No	2488 (95.2)	79 (24.0)	0	2567 (85.0)
Yes	126 (4.8)	255 (76.0)	78 (100.0)	454 (15.0)

Abbreviations: ADL: Activities of Daily Living, IADL: Instrumental Activities of Daily Living.

^a^Cognitive impairment included a Mini-Mental State Examination (MMSE) score of <27 and/or dementia. Multimorbidity is defined as the presence of 3+ diseases without dementia. Characteristics are reported as number (%) or mean ± standard deviation, as appropriate. All sociodemographic, clinical and functional characteristics were fixed at baseline.

The mean age of participants was 74.7 ± 11.2 years. Sixty-three point five per cent were women. At baseline, nursing home residents were older and had a higher burden of disease, functional impairment and disability than those living elsewhere.

Over 15 years, the total number of transitions observed was 24 209. Overall, 1485 (49.2%) participants experienced 1–5 transitions (mean age, 71.1), 720 (23.8%) experienced 5–10 transitions (mean age, 76.0) and 816 (26.7%) experienced >10 transitions (mean age, 75.9). The distribution of care transitions by age group and gender is shown in Table S3. Most participants experienced fewer than 10 transitions (73.0%). Of these, 53.1% were under 78 years (31.7% were women and 21.4% were men), and 19.9% were over 78 years (15.1% and 4.7%, respectively, women and men).

**Figure 1:**
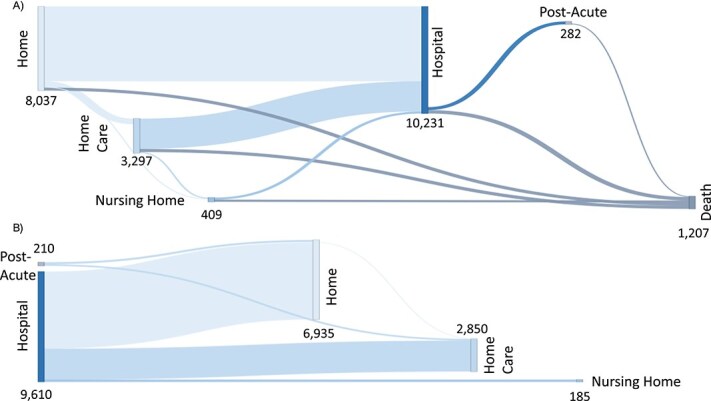
Sankey diagram displaying the total number of transitions across living and care settings observed over 15 years. (A) Forward transitions and (B) backward transitions after discharge from the hospital and postacute care. Note. This figure represents the number of times the transitions occurred in a specific pattern over 15 years, and a single person can contribute more time to the transition pattern. The median follow-up was 11.23 years. The mean follow-up was 9.50 years.

Figure 1 illustrates the number of transitions from and toward each setting in both forward and backward directions. The estimated mean number of transitions through each setting over 15 years was 7.0 for home, 2.9 for home with formal care services, 0.3 for nursing home, 9.8 for hospital and 0.3 for postacute or rehabilitation care, respectively. The mean length of stay was 2.4 years at home, 1.0 years at home receiving home care and 1.4 years in nursing homes. Finally, the mean hospital stay was 8 days, and the mean length of stay in postacute care facilities was 17 days.

Table 2 presents the association between potential clinical and functional characteristics and cumulative volume of transitions observed over 15 years, independent of transition type. Older age, being unpartnered, having multimorbidity and presenting with slow gait speed were significantly associated with more transitions. Conversely, being a woman, having higher education and having dementia or cognitive impairment and disability in ADL and IADL were significantly associated with fewer transitions.

**Table 2:** Association between sociodemographic, clinical and functional characteristics and the number of care transitions across living and care settings.

	Model 1 IRR (95% CI)	Model 2 IRR (95% CI)	Model 3 IRR (95% CI)
Age	1.01 (1.01; 1.01)	1.01 (1.01; 1.01)	1.01 (1.01; 1.01)
Women	0.89 (0.87; 0.92)	0.89 (0.86; 0.91)	0.89 (0.87; 0.91)
High school/university	1.10 (1.06; 1.14)	1.06 (1.02; 1.10)	1.06 (1.03; 1.10)
Unpartnered	1.10 (1.07; 1.13)	1.09 (1.07; 1.12)	1.09 (1.06; 1.12)
Cognitive impairment^a^		0.74 (0.71; 0.77)	0.77 (0.75; 0.80)
Multimorbidity (3+ diseases)^a^		1.26 (1.24; 1.28)	1.25 (1.23; 1.27)
Slow gait speed (<0.8 m/s)			1.05 (1.01; 1.08)
≥1 Impaired ADL^a^			0.66 (0.61; 0.70)
≥1 Impaired IADL^a^			0.88 (0.84; 0.92)

Abbreviations: ADL, Activities of Daily Living; IADL, Instrumental Activities of Daily Living; IRR, incidence rate ratio, 95% CI, 95% confidence interval. Note. Impairments in ≥1 ADL and ≥ 1IADL were added separately because of the collinearity of the variables. The models are mutually adjusted. All sociodemographic, clinical and functional characteristics were fixed at baseline.

^a^Cognitive impairment included a Mini-Mental State Examination (MMSE) score of <27 and/or dementia. Multimorbidity is defined as the presence of 3+ diseases without dementia.

Tables 3 and 4 show the association between participants’ demographic, clinical and functional characteristics and specific transitions across living and care settings. Older age was associated with an increased likelihood of transitioning from less intensive (e.g. home with or without social care) to more intensive (e.g. hospital) settings (HR range 1.06–1.15). Moreover, older participants showed a higher likelihood of being in receipt of home care after discharge from a hospital or a postacute care facility (HR range 1.06–1.08). Women had a higher chance than men of needing home care after discharge from a hospital or post-acute facility (HR range 1.35–4.38). Women were at lower risk of hospitalisation (HR range 0.58–0.80) compared with men. Higher educational attainment and being unpartnered showed similar risk profiles for hospitalisation from home (HRs 1.08 and 1.08, respectively) and for discharged from hospital to home with social services (HRs 1.15 and 1.11, respectively). Participants with cognitive impairment (including those with dementia diagnoses) were at higher risk of nursing home placement (HR range 1.99–2.25), regardless of their living or care setting. Cognitive impairment reduced both the likelihood of hospitalisation (HR range 0.61–0.84) and returning home (HR range 0.88–0.92). Multimorbidity increased risk of receiving home care services for those living at home (HR 1.20) and those discharged from the hospital (HR 1.21) and the risk of being hospitalised for those living at home independently or with home care support (HR range 1.14–1.23). A slower gait was associated with higher hospitalisation risk (HR range 1.11–1.50) and risk of discharge from the hospital to a home with care services (HR 1.21) and to a nursing home (HR 1.48). Finally, a slower gait speed decreased the likelihood of needing postacute care after hospitalisation (HR 0.54). Disability, measured through ADL and IADL impairments, was associated with a higher risk of nursing home admission after hospital discharge (HR 3.07 and HR 2.57, respectively) and a reduced risk of returning home independently (HR 0.43 and HR 0.43, respectively). The competing risk of death was always considered in the associations. Table S4 shows an association between potential characteristics and mortality in different living and care settings. Women, compared with men, showed a lower likelihood of dying in all living and care settings (HR range 0.49–0.63). Older participants, those with cognitive impairment and slow gait, had an increased risk of death at home, with or without formal social care, and in nursing homes (HR range 1.07–1.81).

**Table 3:** Associations between sociodemographic, clinical and functional characteristics and transitions across living and care settings (forward).

Transition Across Settings 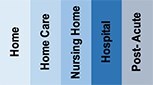										
	**Volume of transitions**	**Age**	**Women**	**High school/University**	**Unpartnered**	Cognitive Impairment*	Multimorbidity (3+ diseases)*	**Slow Gait Speed (<0.8 m/s)**	**≥1 impaidred ADL**	**≥1 impaired IADL**
		HRs (95%CI)	HRs (95%CI)	HRs (95%CI)	HRs (95%CI)	HRs (95%CI)	HRs (95%CI)	HRs (95%CI)	HRs (95%CI)	HRs (95%CI)
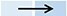	534	**1.15 (1.14; 1.16)**	**1.41 (1.14; 1.74)**	1.10 (0.87; 1.37)	1.19 (0.97; 1.45)	**0.66 (0.52; 0.85)**	**1.20 (1.06; 1.35)**	1.09 (0.89; 1.34)	0.95 (0.50; 1.83)	0.73 (0.51; 1.02)
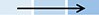	76	**1.14 (1.11; 1.18)**	1.04 (0.61; 1.78)	0.87 (0.51; 1.49)	1.00 (0.59; 1.71)	**2.15 (1.27; 3.62)**	0.84 (0.63; 1.11)	1.58 (0.94; 2.66)	1.75 (0.54; 5.69)	1.15 (0.57; 2.32)
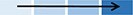	7.317	**1.06 (1.05; 1.06)**	**0.70 (0.66; 0.73)**	**1.08 (1.01; 1.16)**	**1.08 (1.02; 1.13)**	**0.84 (0.78; 0.91)**	**1.23 (1.20; 1.27)**	**1.21 (1.13; 1.29)**	0.99 (0.80; 1.23)	**1.22 (1.10; 1.36)**
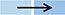	101	**1.07 (1.03; 1.11)**	1.30 (0.71; 2.38)	1.44 (0.89; 2.34)	0.96 (0.56; 1.66)	**1.99 (1.30; 3.07)**	0.85 (0.62; 1.16)	**1.81 (1.14; 2.88)**	0.71 (0.35; 1.42)	1.01 (0.63; 1.64)
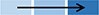	2.895	**1.02 (1.01; 1.03)**	**0.80 (0.73; 0.87)**	1.03 (0.94; 1.12)	1.00 (0.92; 1.09)	0.92 (0.84; 1.01)	**1.14 (1.07; 1.22)**	**1.11 (1.03; 1.21)**	1.05 (0.92; 1.20)	0.98 (0.89; 1.08)
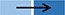	199	0.99 (0.97; 1.01)	**0.58 (0.40; 0.85)**	1.35 (0.93; 1.95)	1.11 (0.77; 1.60)	**0.61 (0.44; 0.86)**	0.95 (0.79; 1.14)	**1.50 (1.05; 2.14)**	0.86 (0.58; 1.27)	1.11 (0.78; 1.56)
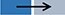	282	**0.95 (0.94; 0.96)**	1.26 (0.98; 1.62)	1.21 (0.83; 1.76)	0.79 (0.62; 1.02)	0.87 (0.57; 1.31)	1.07 (0.93; 1.24)	**0.54 (0.39; 0.76)**	0.40 (0.13; 1.21)	0.76 (0.48; 1.20)

^*^Cognitive impairment included a Mini-Mental State Examination (MMSE) score of <27 and dementia. Multimorbidity is defined as the presence of 3+ diseases without dementia.

Abbreviations: ADL: Activities of Daily Living, IADL: Instrumental Activities of Daily Living, HR: Hazard Ratios, 95%CI: 95% Confidence Interval.

Note. Impairments in ≥1 ADL and ≥1IADL were added separately because of the collinearity of the variables. The model is mutually adjusted. The estimates of the association between potential predictors and death in the examined living and care settings are shown in Table S4.

**Table 4:** Associations between sociodemographic, clinical and functional characteristics and transitions across living and care settings (backward).

Transition Across Settings 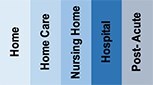										
	**Volume of transitions**	**Age**	**Women**	**High school/University**	**Unpartnered**	Cognitive Impairment*	Multimorbidity (3+ diseases)*	**Slow Gait Speed (<0.8 m/s)**	**≥1 impaidred ADL**	**≥1 impaired IADL**
		HRs (95%CI)	HRs (95%CI)	HRs (95%CI)	HRs (95%CI)	HRs (95%CI)	HRs (95%CI)	HRs (95%CI)	HRs (95%CI)	HRs (95%CI)
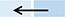	45	**0.95 (0.92; 0.99)**	1.45 (0.67; 3.11)	0.85 (0.40; 1.82)	0.70 (0.38; 1.32)	0.60 (0.23; 1.59)	0.91 (0.61; 1.35)	**0.40 (0.19; 0.86)**	0.64 (0.10; 4.25)	0.66 (0.26; 1.70)
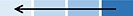	6.721	**0.95 (0.95; 0.95)**	**0.94 (0.89; 0.99)**	1.04 (0.97; 1.12)	**0.81 (0.77; 0.85)**	**0.92 (0.85; 0.99)**	1.00 (0.97; 1.03)	**0.55 (0.52; 0.59)**	**0.43 (0.34; 0.53)**	**0.43 (0.38; 0.48)**
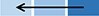	2.704	**1.06 (1.05; 1.06)**	**1.35 (1.22; 1.49)**	**1.15 (1.05; 1.27)**	**1.11 (1.01; 1.22)**	**0.88 (0.80; 0.97)**	**1.21 (1.13; 1.29)**	**1.21 (1.11; 1.32)**	1.04 (0.90; 1.20)	**1.33 (1.20; 1.47)**
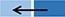	185	**1.05 (1.03; 1.08)**	0.91 (0.62; 1.32)	**2.06 (1.39; 3.04)**	1.42 (0.96; 2.10)	**2.25 (1.63; 3.12)**	**0.65 (0.54; 0.78)**	**1.48 (1.03; 2.14)**	**3.07 (2.12; 4.44)**	**2.57 (1.77; 3.74)**
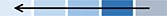	169	**0.96 (0.94; 0.98)**	**0.69 (0.49; 0.96)**	0.91 (0.54; 1.52)	1.03 (0.72; 1.45)	1.17 (0.63; 2.17)	0.99 (0.83; 1.19)	1.13 (0.72; 1.79)	0.57 (0.04; 7.39)	**0.38 (0.18; 0.79)**
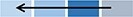	37	**1.08 (1.04; 1.13)**	**4.38 (1.53; 12.54)**	1.33 (0.53; 3.32)	0.92 (0.43; 1.99)	1.03 (0.33; 3.20)	3.06 (0.90; 10.39)	1.01 (0.45; 2.28)	0.09 (0.01; 18.37)	**2.63 (1.12; 6.20)**

^*^Cognitive impairment included a Mini-Mental State Examination (MMSE) score of <27 and dementia. Multimorbidity is defined as the presence of 3+ diseases without dementia.

Abbreviations: ADL, Activities of Daily Livingl IADL, Instrumental Activities of Daily Living, HR, hazard ratios; 95% CI: 95% confidence interval.

Note. Impairments in ≥1 ADL and ≥1IADL were added separately because of the collinearity of the variables. The model is mutually adjusted. The estimates of the association between potential predictors and death in the examined living and care settings are shown in Table S4.

## Discussion

This study identified several individual characteristics that could potentially shape the trajectories of older adults across different living and care settings. Individuals with burdensome medical conditions (i.e. multimorbidity and a proxy of frailty) were observed to be the main contributors to an increasing number of transitions. In contrast, cognitive impairment and disability were associated with fewer transitions. Older age, multimorbidity and slow gait as a proxy for frailty consistently emerged as factors increasing the likelihood of hospitalisation and transition to higher-intensity settings (i.e. home care and nursing home). These characteristics reduced the likelihood of hospital discharge to home. The presence of cognitive impairment, and to some extent disability, strongly impacted the risk of nursing home placement, independent of the initial living setting, but conversely were associated with fewer transitions. Compared with men, women had a reduced risk of hospitalisation and a higher risk of receiving home care, whether they lived at home or were discharged from the hospital. To our knowledge, this is the first study to comprehensively examine the complexity of the journeys of older adults across several living and care settings. Understanding these risk profiles could inform targeted interventions and support systems to optimise transitional care and promote better outcomes for older adults.

Risk factors triggering the use of formal social care have been studied previously. Some studies have found women are more likely to receive home care, potentially because of greater life expectancy, which means they outlive spouses, relatives and friends, eventually limiting access to informal care [32, 40–43]. Some authors have previously investigated the role of disabilities in accessing home care [31, 41–43]. In line with previous studies, impairments in IADL, rather than ADL, were the main trigger for receiving home care services. Beyond these confirmatory results, our study established a relationship between multimorbidity and poor physical function (i.e. slow gait) and transition to home care services from home or at hospital discharge. This observation reinforces the general agreement on incorporating measures of disease burden and physical function, such as walking speed, as indicators of the need for formal home care [7, 40–43].

Previous research has identified several predictors of nursing home admission in older individuals living at home, including age, cognitive impairment, slower walking speed, multiple chronic conditions and poor performance in ADL and IADL [14–16, 44]. Cognitive impairment and dementia have been shown to pose potential risks associated with nursing home placement, reinforcing the significant impact of cognitive health on admission to nursing home [15, 16, 44]. A decline in cognitive capacity can significantly affect an individual’s ability to live independently and perform daily tasks, leading to an increased likelihood of nursing home admission. Studies have also shown that lower mobility can have an impact on nursing home admission and often co-occurs with cognitive decline, implying a relationship [14, 33, 45]. Overall, in our study, we augmented the existing body of knowledge by underscoring the complex interplay between age, cognitive health and physical mobility in determining the pathway toward nursing home care.

Our findings indicate advanced age, being a man, multimorbidity, ADL impairment and slow gait are associated with an increased likelihood of hospitalisation. These associations may be attributed to the cumulative burden of age-related health conditions and the increased need for medical interventions among older adults. Chamberlain *et al.* found that multimorbidity (defined as more than or equal to three diseases), both alone and combined with functional limitation, was associated with increased hospitalisation risk [13, 26, 46]. This suggests that hospitalisation is influenced by a complex interplay between individual, functional and multimorbidity burdens. However, previous studies have primarily focused on how multimorbidity impacts hospitalisation, focusing exclusively on clinical aspects, while overlooking the broader context of individual function and other contributors. The novelty of our study lies in hospitalisation as a competing risk in a complex journey, emphasising the frequency of transitions to hospital and how various predictors differ based on the initial setting. Walking speed was strongly associated with hospitalisation risk [28, 47]. For instance, Montero-Odasso *et al.* confirmed that walking speed could serve as a discriminative factor for hospitalisation risk in older adults, as it likely indicates a complex interplay of impairments preceding clinical manifestation of conditions such as frailty or cognitive decline [47]. Additionally, it is important to recognise that a considerable number of hospitalisations among older adults can be prevented through timely and comprehensive management of chronic diseases [48, 49]. We found that low gait speed, as a proxy for frailty, decreased the likelihood of receiving postacute care following hospitalisation, potentially due to lower perceived rehabilitation potential in frailer individuals with lower mobility function [50]. However, these associations are not well established. Previous studies have focused on the association between lower gait speed and adverse health outcomes or specific diseases such as stroke or hip fracture [28, 47, 51]. This may be due to the difficulty in capturing postacute or rehabilitation care and differentiating it from the rest of hospitalisations.

Consistent with our results, the absence of cognitive impairment has been consistently found to be a significant predictor of discharge home [52, 53]. Younger individuals were also more likely to return home after hospitalisation, potentially due to their lower prevalence of chronic health conditions or stronger social support networks that facilitate recovery and care at home. By considering these factors during discharge planning, appropriate support and resources can be provided to facilitate successful transitions and optimise patient outcomes [54, 55].

An in-depth analysis of older adults’ journeys through health and social care services revealed a multifaceted path of experiences that they encounter across different complexities of living and care settings. An increasing number of studies have focused on the need for a care continuum among older adults, which considers the complexity of individuals, including clinical and functional characteristics. These studies highlight the need to shift toward integrated and patient-centred models to meet the unaddressed needs of this population [56–58]. This is a key finding, as our results indicate that considering the whole journey and having an overview of associated characteristics can deepen our understanding of social and health system intricacies. However, further research is needed to further explore these results and examine how healthcare experiences differ among diverse populations.

This study had several strengths. Our study involved an exhaustive analysis of the health and social care system journeys of older adults, considering transitions between different living and care settings. This study used a well-established cohort of Swedish adults to collect reliable data over a long follow-up period. Data were linked to administrative records to increase the robustness of the study and to provide valuable insights into trends, patterns and associations. The main limitation was the memorylessness assumption of the model, which implies that the probability of transitioning to a state is determined by the current state rather than past states. Another limitation of that the results may not be generalisable to care transition patterns involving fewer than 20 transitions, as the analysis primarily reflects individuals with a higher number of transitions across states. Additionally, our study did not consider the progression of clinical and functional characteristics throughout the follow-up period.

In conclusion, our study highlights the complexity of care transitions experienced by older adults and identifies several factors associated with them. While older age, a higher disease burden and mobility impairment increase the transitions older individuals experience toward the hospital and long-term care, the presence of cognitive and physical disabilities reduces the likelihood of being hospitalised and increases the chance to receive social support at home and be placed in a nursing home. As such, different clinical and functional profiles, if correctly and timely assessed in a comprehensive manner, might open the possibility of designing personalised care pathways aimed at reducing the excessive and burdensome utilisation of healthcare services.

## Supplementary Material

aa-24-1116-File002_afaf006
