# Supplementary material for: Predictors of 15-year transitions across living and care settings in a population of Swedish older adults
Source: Age Ageing. 2025 Jan 26;54(1):afaf006. doi: 10.1093/ageing/afaf006 (PMC11766743; doi:10.1093/ageing/afaf006)
Supplement: aa-24-1116-File002_afaf006 [file aa-24-1116-file002_afaf006.docx]

**Predictors of 15-year transitions across living and care settings in a population of Swedish older adults**

## Supplementary

**Appendix 1. The Swedish National Study on Aging and Care in Kungsholmen**

Initially, a random sample of 5,111 individuals was selected, of which 89.8% were alive and eligible for the study (Fig. S1). Of the 4,590 eligible participants, 1,227 declined to participate, accounting for 27% of the eligible sample. Consequently, the final study sample for the baseline SNAC-K consisted of 3,363 participants.


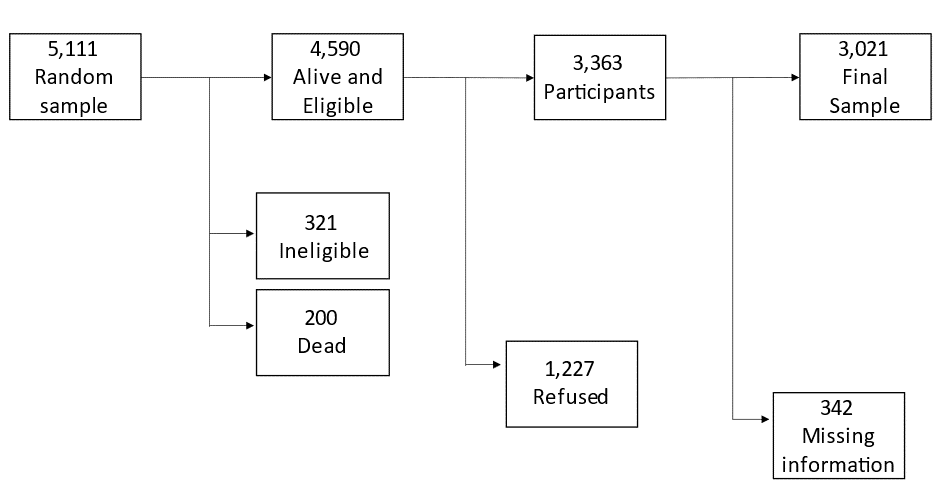


**Figure S1.** Flow chart of the study population

**Note.** Ineligible: Deaf/ Language/ Moved + No contact information
Refused:  Proxy refused + Participant refused + Withdrew

The SNAC-K study utilized a stratified sampling approach, initially dividing the population into distinct age groups and then selecting a random sample from each group. This study included 11 age cohorts determined using two different age intervals (Fig. S2):6-year intervals for younger cohorts (60, 66, 72, and 78 years) and 3-year intervals for older cohorts (81, 84, 87, 90, 93, 96, and 99+ years). Follow-up assessments were conducted every 6 years for younger cohorts (aged 60-78) and every 3 years for older cohorts (aged ≥ 78 years). The mean follow-up time for the cohort was 9.5 years, with a range of 0.05-15.8 years.


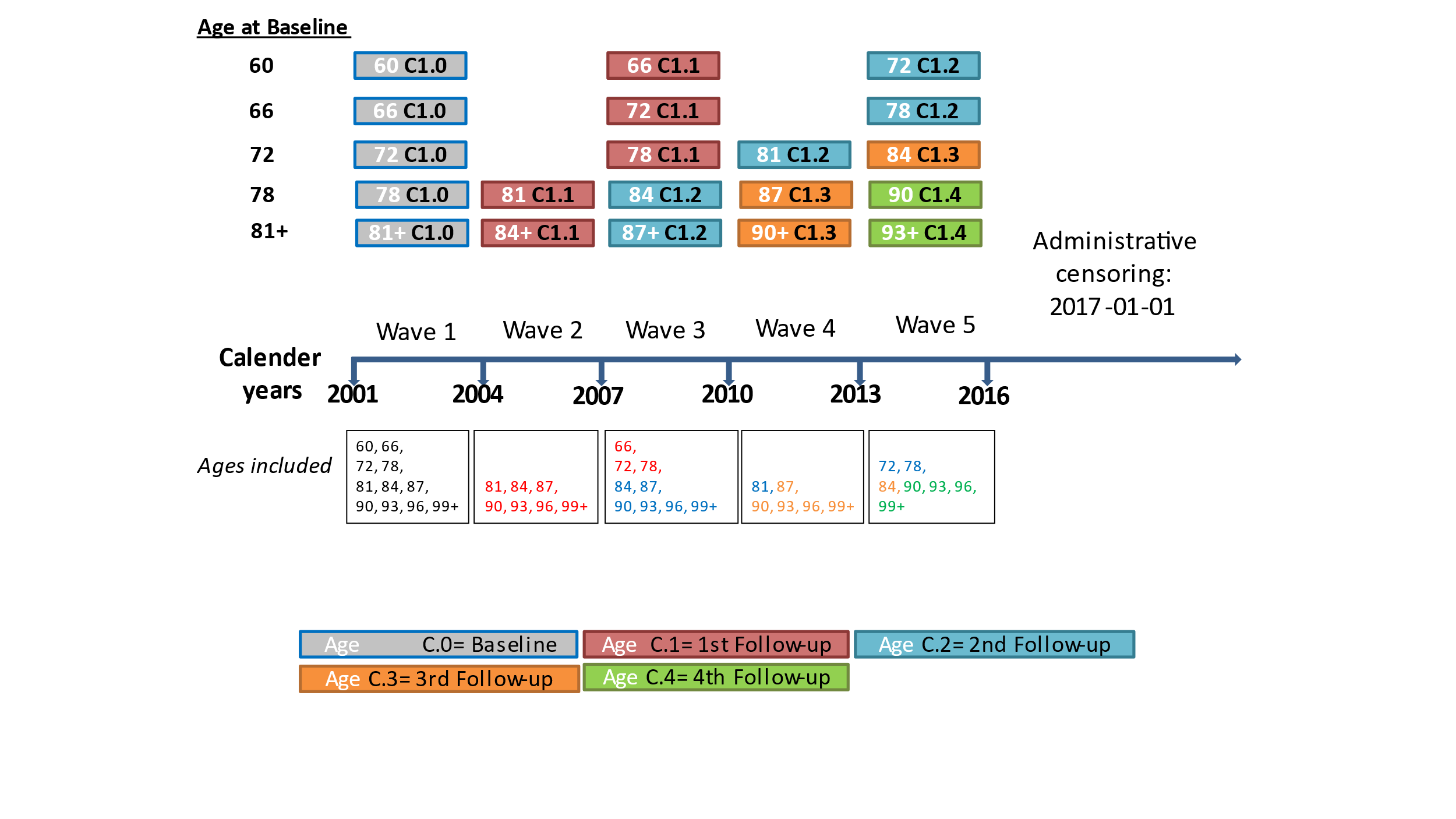


**Figure S2.** Distribution of sample age cohorts (SNAC-K) for baseline and follow-up

Following the SNAC-K protocols, participants underwent thorough clinical and functional evaluation by trained doctors, nurses, and psychologists in each study wave. In addition, home visits were made to those who agreed to participate but could not visit the research center or people living in nursing homes. The assessment process involved face-to-face interviews, medical record review, physical tests, and questionnaires. Any participant or representative who declined participation, could not be contacted, moved away from the study area, or missed an entire evaluation, with no further evaluations available, was classified as being lost to follow-up.

The Karolinska Institute Ethics Committee and Regional Ethical Review Board of Stockholm approved all SNAC-K waves involved in this study, including their use and integration with Swedish register data. All participants or caregivers of the patients with cognitive impairment provided written informed consent.

**Table S1.** Demographic information of 27% of the participants who refused to participate compared with SNAC-K population

|  | Refused to participate | | | SNAC-K population | | |
| --- | --- | --- | --- | --- | --- | --- |
| Age group | Gender | | Total | Gender | | Total |
|  | Men (%) | Women (%) | N= 1,227 | Men (%) | Women (%) | N= 3,363 |
| 60-69 years | 181 (44.8) | 223 (55.2) | 404 | 569 (43.6) | 735 (56.7) | 1304 |
| 70-79 years | 136 (35.1) | 252 (64.9) | 388 | 340 (36.5) | 592 (63.5) | 932 |
| 80-89 years | 84 (24.0) | 266 (76.0) | 350 | 187 (29.2) | 454 (70.8) | 641 |
| ≥ 90 years | 13 (15.3) | 72 (84.7) | 85 | 85 (17.5) | 401 (82.5) | 486 |

The results of the two-sample t-tests indicated that there was no statistically significant difference in the mean distribution for both gender and age between the sample (N=3,363) and refusal at baseline (N=1,227, 27%). The p-values for gender and age were 0.3842 and 0.09213, respectively.

**Table S2.**Sociodemographiconal characteristics according to baseline living settings of the excluded participants compared to the included participants

|  | **Excluded Participants** | | | | **Included participants** | | | |
| --- | --- | --- | --- | --- | --- | --- | --- | --- |
|  | **Home**  **n (%)** | **Home Care**  **n (%)** | **Nursing Home**  **n (%)** | **Tot**  **N (%)** | **Home**  **n (%)** | **Home Care**  **n (%)** | **Nursing Home**  **n (%)** | **Tot**  **N (%)** |
| **Sample** | 108 (31.6) | 114(33.3) | 120(35.1) | 342 (100.0) | 2,614 (86.5) | 329 (10.8) | 78 (2.7) | 3,021 (100.0) |
| **Age (years)** |  |  |  |  |  |  |  |  |
| Mean ± SD |  |  |  | 84.67 ± 0.56 |  |  |  | 74.7 ± 11.2 |
| 60-69 years | 33 (30.6) | 6 (5.3) | 1(0.8) | 40 (11.8) | 1,252 (47.9) | 11 (3.3) | 1 (1.4) | 1,264 (41.8) |
| 70-79 years | 22 (20.4) | 20 (17.5) | 16 (13.3) | 58 (16.8) | 822 (31.5) | 53 (16.2) | 6 (7.6) | 881 (29.2) |
| 80-89 years | 28 (25.9) | 34 (29.8) | 29 (24.2) | 91 (26.7) | 413 (15.8) | 114 (34.6) | 16 (20.5) | 543 (17.9) |
| ≥ 90 years | 25 (23.1) | 54 (47.4) | 74 (61.7) | 153 (44.7) | 127 (4.8) | 151 (45.9) | 55 (70.5) | 333 (11.1) |
| **Gender** |  |  |  |  |  |  |  |  |
| Man | 39 (36.1) | 26 (22.8) | 14 (11.7) | 79 (23.1) | 1,022 (39.1) | 66 (20.1) | 14 (17.9) | 1,102 (36.5) |
| Women | 69 (63.9) | 88 (77.2) | 106 (88.3) | 263 (76.9) | 1,592 60.9) | 263 (79.9) | 64 (82.1) | 1,919 (63.5) |

**Appendix 2. Markov Multistate model**


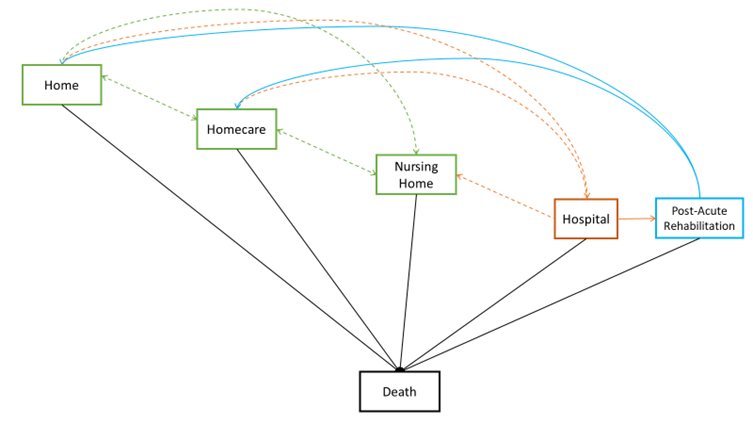


**Figure S3** Outcomes of the Markov Multistate Transition Model

A Markov multistate reversible model was employed to determine how the likelihood of transitioning between different care settings was related to participants’ physical, cognitive, and functional characteristics. We employ this model to address these three major issues.

(i) Recognizing transitions across various care providers based on their underlying characteristics.

(ii) Addressing the Reversible Model. In multistate models, a reversible model refers to a specific type of model structure, in which transitions between different states are allowed to occur in both directions. This means that an individual can transition from one state to another, and can also transition back to the previous state, which means that the transitions are not strictly unidirectional.

(iii) employing several outcomes simultaneously (i.e., hospitalization, institutionalization, use of formal care, and mortality).

Figure S3 graphically depicts the proposed model and the possible transitions. The transition occurs between different care settings in this model, including the entry states (home, home care, and nursing home), intermediate states (hospital and post-acute/rehabilitation), and the absorbing state (death).

In the context of a Markov multistate reversible model, Hazard Ratios (HRs) are often used to compare hazard rates or the likelihood of transitioning from one state to another. HRs represent the instantaneous rate of transitioning from one state to another at any given time, conditional on the system being in a particular state at that time. An HR of 1 indicates that there is no difference. An HR greater than 1 indicates that the transition likelihood is higher in the group of interest. An HR of less than 1 suggests a lower transition likelihood for the group of interest. In a Markov multistate model, the HR describes the relative likelihood of moving between different states, such as from living independently to entering a care facility, given different factors like age, health status, or other covariates. Moreover, in multi-state models, hazard ratios represent the relative risk of transitioning to a particular outcome compared to all other possible outcomes from the current state. Therefore, the reference is not limited to a single category (e.g., discharge home without social care) but includes all other possible transitions.

The goodness of fit of the model was assessed by comparing the observed and expected prevalence in each state.

A limitation of this model was the use of two distinct time scales inherent to SNAC-K: one tracking the hospitalization period based on the calendar (i.e., derived from administrative data sources), and the other tracking the follow-up period for 3 or 6 years (i.e., inherent to the SNAC-K design). To address this issue, we used age as the timescale.

**Table S3.** Transitions group stratifies by gender and age group

|  | **Age** | | | | | |
| --- | --- | --- | --- | --- | --- | --- |
|  | **60-69 years**  **n (%)** | **70-79 years**  **n (%)** | **80-89 years**  **n (%)** | **≥ 90 years**  **n (%)** | **Total N (%)** | **Mean ± SD** |
| **From 1 to 5** |  |  |  |  | 1485 (49.2) | 71.1 ± 11.0 |
| **Men** | 345 (42.3) | 115 (33.9) | 47 (27.0) | 29 (18.5) | 536 (36.1) |  |
| **Women** | 470 (57.7) | 224 (66.1) | 127 (73.0) | 128 (81.5) | 949 (63.9) |  |
| **From 6 to 10** |  |  |  |  | 720 (23.8) | 76.0 ± 10.8 |
| **Men** | 104 (44.3) | 83 (38.4) | 45 (28.0) | 22 (20.4) | 254 (35.3) |  |
| **Women** | 131 (55.7) | 133 (61.6) | 116 (72.0) | 86 (79.6) | 466 (64.7) |  |
| **From 11 to 15** |  |  |  |  | 365 (12.1) | 76.2 ± 9.5 |
| **Men** | 48 (46.2) | 53 (43.4) | 32 (32.0) | 7 (17.9) | 140 (38.4) |  |
| **Women** | 56 (53.8) | 69 (56.6) | 68 (68.0) | 32 (82.1) | 225 (61.6) |  |
| **From 16 to 20** |  |  |  |  | 207 (6.8) | 76.4 ± 9.1 |
| **Men** | 24 (48.0) | 30 (34.5) | 22 (42.3) | 3 (16.7) | 79 (38.2) |  |
| **Women** | 26 (52.0) | 57 (65.5) | 30 (57.7) | 15 (83.3) | 128 (61.8) |  |
| **From 21 to 30** |  |  |  |  | 163 (5.4) | 75.8 ± 8.2 |
| **Men** | 18 (51.4) | 30 (39.5) | 13 (30.2) | 1 (11.1) | 62 (38.0) |  |
| **Women** | 17 (48.6) | 46 (60.5) | 30 (69.8) | 8 (88.9) | 101 (62.0) |  |
| **More than 31** |  |  |  |  | 81 (2.7) | 73.1 ± 8.4 |
| **Men** | 13 (52.0) | 14 (34.1) | 4 (30.8) | - | 31 (38.3) |  |
| **Women** | 12 (48.0) | 27 (65.9) | 9 (69.2) | 2 (100.0) | 50 (61.7) |  |

**Table S4.** Association between demographic, clinical, and functional characteristics and transitions across living and care settings and death

Cognitive impairment included a Mini-Mental State Examination (MMSE) score of <27 and dementia. Multimorbidity was defined as the presence of 3 diseases without dementia. Abbreviations: ADL: Activities of Daily Living, IADL: Instrumental Activities of Daily Living.
